# Barriers to horizontal cell transformation by extracellular vesicles containing oncogenic H-*ras*

**DOI:** 10.18632/oncotarget.10627

**Published:** 2016-07-16

**Authors:** Tae Hoon Lee, Shilpa Chennakrishnaiah, Brian Meehan, Laura Montermini, Delphine Garnier, Esterina D'Asti, Wenyang Hou, Nathalie Magnus, Tenzin Gayden, Nada Jabado, Kolja Eppert, Loydie Majewska, Janusz Rak

**Affiliations:** ^1^ Research Institute of the McGill University Health Centre, Glen Site, McGill University, QC, H4A 3J1 Canada

**Keywords:** exosomes, extracellular vesicles, horizontal transformation, oncogenes, ras

## Abstract

Extracellular vesicles (EVs) enable the exit of regulatory, mutant and oncogenic macromolecules (proteins, RNA and DNA) from their parental tumor cells and uptake of this material by unrelated cellular populations. Among the resulting biological effects of interest is the notion that cancer-derived EVs may mediate horizontal transformation of normal cells through transfer of mutant genes, including mutant *ras*. Here, we report that H-*ras*-mediated transformation of intestinal epithelial cells (IEC-18) results in the emission of exosome-like EVs containing genomic DNA, HRAS oncoprotein and transcript. However, EV-mediated horizontal transformation of non-transformed cells (epithelial, astrocytic, fibroblastic and endothelial) is transient, limited or absent due to barrier mechanisms that curtail the uptake, retention and function of oncogenic H-*ras* in recipient cells. Thus, epithelial cells and astrocytes are resistant to EV uptake, unless they undergo malignant transformation. In contrast, primary and immortalized fibroblasts are susceptible to the EV uptake, retention of H-*ras* DNA and phenotypic transformation, but these effects are transient and fail to produce a permanent tumorigenic conversion of these cells *in vitro* and *in vivo*, even after several months of observation. Increased exposure to EVs isolated from H-*ras*-transformed cancer cells, but not to those from their indolent counterparts, triggers demise of recipient fibroblasts. Uptake of H-*ras*-containing EVs stimulates but fails to transform primary endothelial cells. Thus, we suggest that intercellular transfer of oncogenes exerts regulatory rather than transforming influence on recipient cells, while cancer cells may often act as preferential EV recipients.

## INTRODUCTION

Pathways of intercellular communication and molecular exchange represent an emerging frontier in confronting the complex aetiology and intractability of many human cancers [1]. Indeed, multiple mechanisms involved in pathological connectivity between cancer cells include paracrine interactions [2], physical contacts, formation of junctions, tunneling nanotubes (TNTs), microtubes [3, 4] and trafficking of signals through the exchange of extracellular vesicles (EVs) [5]. In the latter case, complex molecular assemblies of bioactive molecules, including proteins and nucleic acids, become encapsulated in membrane structures and released from ‘donor’ cells as heterogeneous EV subtypes, including exosomes, microvesicles (MVs) or apoptotic bodies (ABs) [5]. The uptake of EVs by various ‘recipient’ cells and intercellular transfer of their molecular content (cargo) triggers a multitude of biological responses, many implicated in cancer progression [6–9].

Oncogenic pathways play multiple roles in EV-mediated cellular communication, including impact on EV biogenesis, molecular composition, release and especially by virtue of EV-mediated emission of active oncoproteins and transforming nucleic acids themselves [6, 8–11]. Notably, the uptake of tumor-related EVs by recipient cells was found to elicit features reminiscent of malignant transformation, such as altered signalling, changes in morphology, angiogenic phenotype and increase in the clonogenic growth potential [6, 12].

Indeed, the notion of horizontal cellular transformation of normal cells has been raised recently, as a tantalizing implication of the intercellular trafficking of oncogenic macromolecules via EV-dependent and independent mechanisms. According to this paradigm, oncogenic hits need not accumulate exclusively within the genome of a cancer cell clone [13], but instead could propagate across cancer and normal cell populations by the exchange of molecular cargo, especially mutant DNA [10]. The resulting tumorigenic conversion of normal cells [14] could occur either locally (oncogenic ‘field effects’) [15, 16] or at distant organ sites (‘genometastasis’) [17], resulting in accelerated disease progression. In support of this possibility, apoptotic EVs were found to mediate transfer of genomic DNA (gDNA) containing mutant H-*ras*, *Myc* and viral oncogenes, a process that triggered tumor formation by otherwise non-tumorigenic normal rodent fibroblasts [10, 14]. Also, EVs emitted by viable aggressive breast cancer cells were shown to contain transforming proteins [18] or microRNA [19] whose intercellular transfer engendered a fully tumorigenic phenotype in the case of normal fibroblasts or breast epithelial cell recipients, respectively. Similarly, EVs from BCR-ABL-driven leukaemia caused malignant conversion of normal myeloid cells [20–22].

Although horizontal transformation of normal cells represents an intriguing possibility, it also challenges some of the key tenets of the current cancer progression paradigm, such as the genetic lineage and histological continuity between primary and metastatic malignancies [23]. In the present study, we sought to explore this question using a paradigmatic model of cellular transformation and EV-mediated intercellular transfer of the oncogenic H-*ras*. We document that several biological barriers protect normal cells from horizontal transformation by extracellular trafficking of the H-*ras* oncogene. Indeed, intestinal epithelial cells and astrocytes exhibit poor EV uptake, which dramatically increases following malignant transformation. While non-transformed mesenchymal cells readily incorporate oncogenic EVs and acquire the transformed phenotype, these responses are limited and transient in nature. Finally, *in vivo* exposure of non-transformed cells to sources of extracellular oncogenes fails to accelerate tumor formation. We postulate that while extracellular oncogenes (and EVs) are bioactive, their horizontal transformation potential is limited.

## RESULTS

### Enforced expression of mutant H-*ras* in epithelial cells leads to the emission of altered extracellular vesicles containing genomic DNA and H-*ras* oncogene

**Figure 1:**
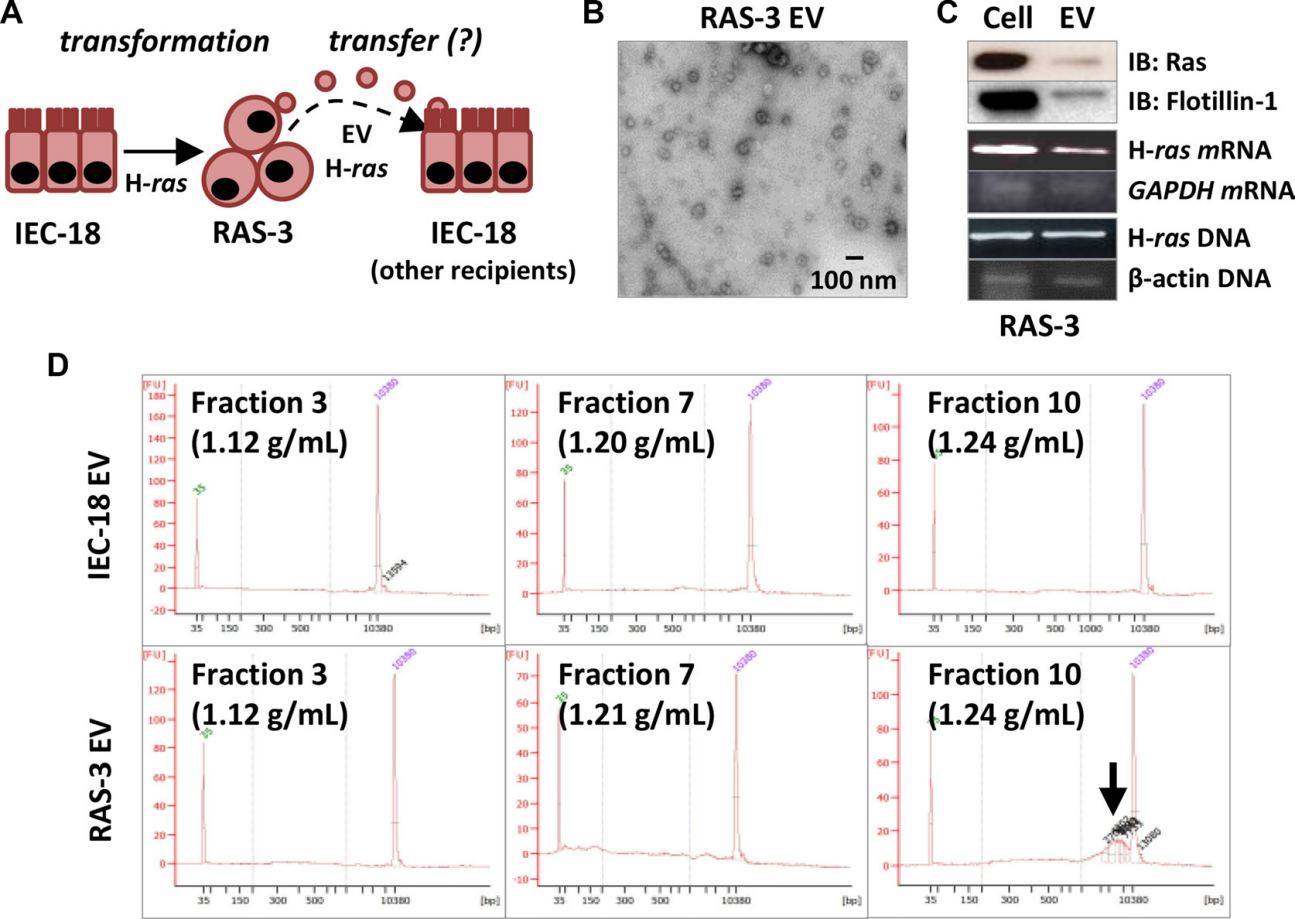
Mutant H-*ras*-mediated cellular transformation stimulates EV-mediated emission of the H-*ras* oncogene and extracellular genomic DNA (**A**) Experimental model: rat epithelial cells (IEC-18) give rise to their transformed clone (RAS- 3) following transformation with human oncogenic H-*ras*. RAS-3 cells emit H-*ras*-containing EVs, which are hypothesized to transfer oncogenic cargo to non-transformed recipient cells, including IEC-18. (**B**) RAS-3 cells emit exosome-like EVs (TEM at 30K magnification). (**C**) RAS-3-derived EVs contain HRAS protein, mRNA and human oncogenic H-*ras* DNA sequences. (**D**) Bioanalyzer QC plots of EV preparations from IEC-18 and RAS-3 cells, resolved on the continuous sucrose gradient. Notable the emission of gDNA in the exosomal fraction 10 (Arrow) of RAS-3-derived EVs.

RAS oncogenes exhibit potent transforming effects demonstrated in a wide range of susceptible target cells *in vitro* and *in vivo* [24, 25]. We first interrogated the potential for EV-mediated horizontal RAS transfer and transformation by testing the properties of the isogenic model system in which non-tumorigenic, phenotypically normal, immortalized rat epithelial cells (IEC-18) gave rise to highly transformed, angiogenic and tumorigenic clonal variant (RAS-3) following enforced expression of the human V12 H-*ras* oncogene [26]. While both IEC-18 and RAS-3 cells produce ample numbers of small exosome-like EVs that pass through 0.2 micrometer pore size filters, this process is markedly enhanced in the case of RAS-3 cells [11]. Moreover, unlike their parental counterparts, RAS-3 cells also incorporate gDNA into their EV cargo, including full-length human mutant H-*ras* sequences [11], along with the corresponding mRNA and HRAS oncoprotein (Figure 1A–1D). These observations suggest that H-*ras* transformation is associated with extracellular emission of potentially oncogenic macromolecules.

### Cellular transformation abrogates indolent cell resistance to the uptake of extracellular vesicles containing oncogenic H-*ras*

**Figure 2:**
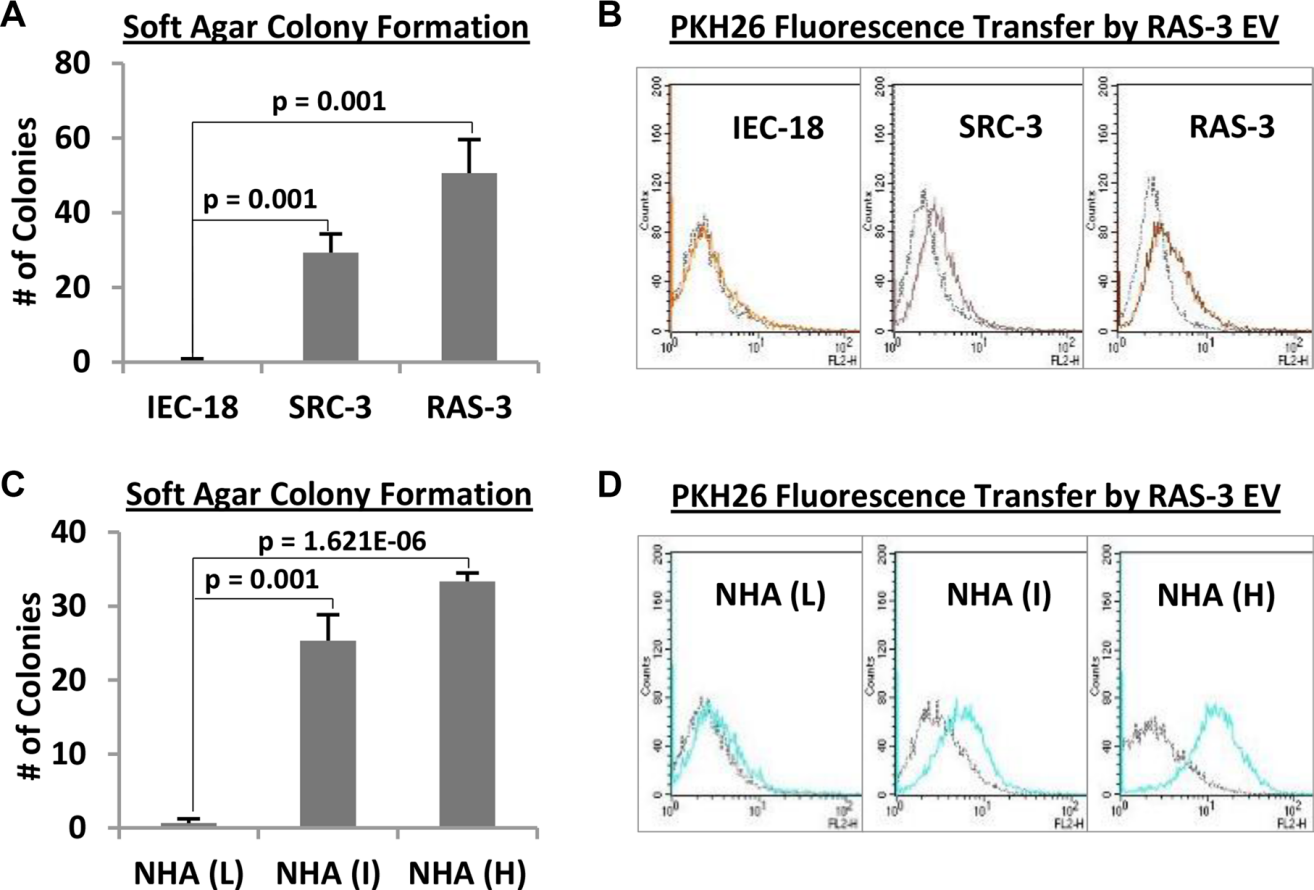
Cellular transformation overcomes resistance of immortalized epithelial cells and astrocytes to the uptake of H-*ras*-containing EVs (**A**) The non-transformed phenotype of IEC-18 cells is revealed by their inability to form colonies in soft agar, a property that contrasts with a robust transformation of their derivatives harboring v-*src* (SRC-3) or H-*ras* (RAS-3) oncogenes. (**B**) IEC-18 cells are unable to efficiently take up PKH26-labelled EVs from RAS-3 cells, while their transformed SRC-3 and RAS-3 variants exhibit robust EV uptake (FACS – PKH26). (**C**) Normal human astrocytes (NHA) undergo spontaneous transformation in serial culture, through low (L), intermediate (I) and high (H) passage numbers, a change revealed by their rising soft agar colony formation ability. (**D**) Transformation of NHA cells is coupled with the increase in their ability to take up EVs produced by PKH26-labelled RAS-3 cells (FACS – PKH26). Data are presented as the means ± SD and are representative of three independent experiments. *P*-values as indicated.

We asked whether the exposure of IEC-18 cells to H-*ras*-containing EVs emanating from their isogenic RAS- 3 counterparts could lead to cellular transformation in a manner reminiscent of experimental H-*ras* transfection (Figure 1A). Surprisingly, incubation of IEC-18 cultures with RAS-3-derived EVs elicited no morphological change, and no biological responses or transfer of H-*ras* gDNA (Supplementary Figure S1; data not shown). Moreover, when RAS-3-derived EVs were pre-labelled with the fluorescent dye (PKH26) [11] and incubated with IEC-18 cultures, virtually no intercellular transfer of membrane fluorescence was registered using FACS analysis (Figure 2). These observations suggest that non-transformed IEC-18 cells are resistant to the uptake of exogenous tumor-related EVs, and thereby to EV-mediated transfer of oncogenic H-*ras* and horizontal transformation.

In contrast to IEC-18 cells, their tumorigenic clonal sublines harboring either oncogenic H-*ras* (RAS- 3) or v-*src* (SRC-3) avidly take up fluorescent EVs, as documented by FACS (Figure 2A and 2B). Similarly, while immortalized, early passage, non-transformed normal human astrocytes (NHA) exhibit minimal uptake of RAS-3-derived fluorescent EVs, the progressive spontaneous transformation of these cells in serial culture leads to a dramatic increase in the retention of EV-associated fluorescence (Figure 2C and 2D). A robust EV uptake is also observed in the case of human glioma (U373 and U87) [6] and medulloblastoma cell lines (DAOY; Supplementary Figure S2).

The mechanism of H-*ras*-mediated increase in the EV uptake by RAS-3 cells remains presently unclear. This property is unrelated to soluble factors (growth factors, enzymes) released by RAS-3 cells, and implicated in the expression of their transformed phenotype [26], as IEC-18 cells remain unable to take up RAS-3 EVs in the presence of RAS-3 conditioned medium (Supplementary Figure S3). Out of several proposed EV uptake mechanisms [27], MAPK-regulated endocytosis [28] and macropinocytosis dependent on the Na+/H+ exchanger (NHE) may be regulated by oncogenic RAS [29, 30]. However, we were unable to abolish the EV uptake by RAS-3 cells using the MEK/MAPK (PD98059) inhibitor (Supplementary Figure S4), and our data with the NHE (EIPA) inhibitor were inconclusive (data not shown). Collectively, our observations suggest that cellular transformation sensitizes certain types of cancer cells to the EV-mediated communication through mechanisms that may involve the activation of RAS and SRC pathways.

### Transient horizontal transformation of mesenchymal cells exposed to oncogenic extracellular vesicles

**Figure 3:**
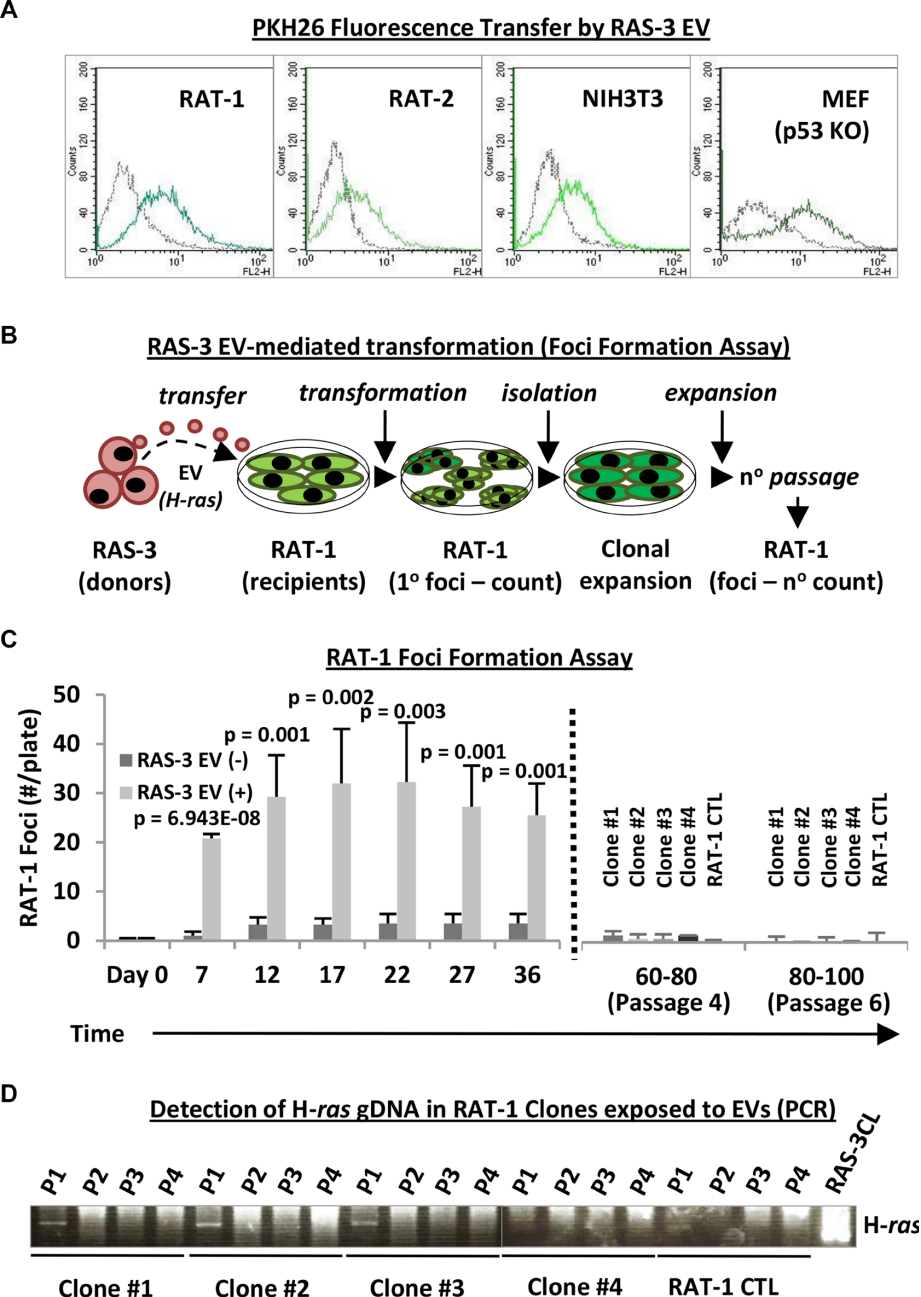
Uptake of mutant H-*ras*-containing EVs by fibroblastic cell lines leads to transient transformation and temporary retention of the exogenous DNA (**A**) Fibroblastic cells, such as RAT-1, RAT-2, NIH3T3 and MEFp53−/− (MEF p53KO), readily take up RAS-3-derived EVs (FACS – PKH26). (**B**) Experimental design for testing transforming potential of RAS-3-derived EVs containing mutant H-*ras* against RAT-1 recipient cells. (**C**) Time-dependent foci formation by RAT-1 fibroblasts in the presence or absence of RAS-3-derived EVs. After 36 days, the remaining foci were individually re-plated (dotted line), and the cells expanded and passaged 6 times (80–100 days), resulting in gradual disappearance of their foci-forming ability. (**D**) The transient retention of exogenous H-*ras* gDNA by EV recipient, RAT- 1 foci-forming cells. The human-specific PCR signal is only observed at passage 1 (P1) post-isolation and later lost in subsequent passages (P2-P4). Data are represented as the means ± SD and are representative of four independent experiments. *P*-values as indicated.

The aforementioned restrictions in the uptake of H-*ras*-containing EVs by epithelial and astrocytic cells were not observed in the case of mesenchymal EV recipients, such as cultured fibroblasts and endothelial cells [11] (Figure 3). Indeed, we observed a robust uptake of fluorescent RAS-3-derived EVs by immortalized fibroblastic cell lines of rat (RAT-1 and RAT-2) and mouse (NIH3T3) origin, and by primary cultures of mouse embryonic fibroblasts from p53-deficient mice (MEFp53−/−) (Figure 3A), all of which lack tumor suppressor mechanisms and are susceptible to oncogenic transformation [31].

We have earlier documented the EV-mediated uptake and retention of H-*ras* gDNA in RAT-1 cells for up to 30 days in culture [11]. Therefore, we first interrogated these cells for the evidence of horizontal transformation (Figure 3A–3D) using foci formation assay [32] (Figure 3B). Remarkably, RAT-1 monolayers incubated with H-*ras*-containing EVs underwent a profound morphological change involving formation of numerous dome-shaped foci, the number of which increased during the first 1–3 weeks post-treatment (Figure 3C; Supplementary Figure S5). Only a small number of spontaneous foci appeared in cultures of untreated RAT- 1 cells, or in the presence of EVs purified from IEC-18 supernatants (Figure 3C; data not shown).

Surprisingly, rather than expanding over time, the numbers of RAT-1 foci began to decline after 3–5 weeks of continued culture. The remaining foci were subsequently isolated, and the cells were re-plated, expanded and cultured for up to 4–6 serial passages, an equivalent of approximately 100 days in culture, at which point their ability to form transformed foci was no longer detectable (Figure 3B and 3C). In keeping with these observations, human H-*ras* gDNA signal was initially readily detectable in 3 out of 4 isolated foci-forming RAT-1 cell clones at the time of their first passage (36 days post-EV treatment - P1; Figure 3D) [11]. However, this signal disappeared completely during subsequent passages *in vitro* (P2-P4). These observations suggest that while EV-derived human H-*ras* DNA enters RAT-1 recipients, it does not fully integrate, amplify or assume its permanently transforming activity.

**Figure 4:**
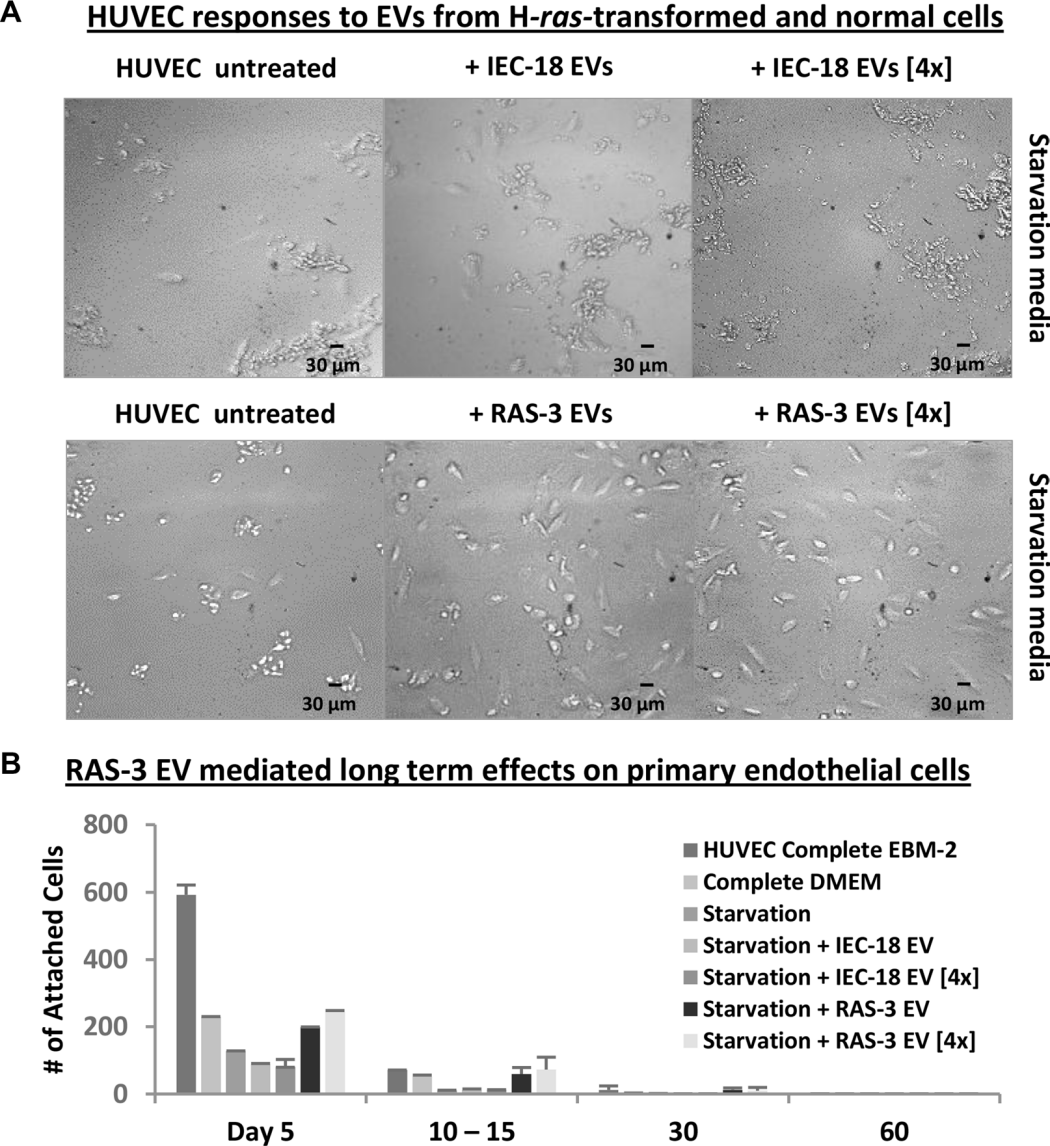
Uptake of mutant H-*ras*-containing EVs by primary endothelial cells leads to a transient growth/survival stimulation in the absence of long-term transformation (**A**) HUVECs require angiogenic factors and serum for long-term survival, which is compromised under growth factor and nutrient starvation conditions (Starvation Media). In this setting, HUVECs lose morphological integrity and viability within 5 days. Addition of purified EVs from RAS-3 cultured cells, which are angiogenic and harbor mutant H-*ras* oncogene, partially reverses this process, while EVs from parental IEC-18 cells lack this activity. Images were taken within 5 days at 200x magnification. (**B**) Transient responses of HUVEC to oncogenic EVs. The adherent HUVECs were counted in the presence of complete or starvation medium, and the effects of RAS-3-derived EVs (containing H-*ras*) were compared to those of IEC-18-derived EVs. The pro-survival effects of EVs were weaker than those of recombinant growth factors and eventually lost, resulting in no evidence of transformed cells. Data are represented as the means ± SD and are representative of two independent experiments.

We have also tested the impact of oncogenic EVs on cultures of primary human endothelial cells (HUVEC). Endothelial cells are naturally exposed to circulating EVs *in vivo* and have been shown to exhibit biological responses to horizontal transfer of oncoproteins and nucleic acids [33]. Therefore, we exposed HUVECs to either RAS-3-derived EVs or controls isolated from IEC-18 cultures and examined cell survival, growth and morphology. Interestingly, RAS-3 EV treatment provoked a change in endothelial cell morphology reminiscent of that induced by angiogenic factors (Figure 4A) [34], including a transient increase in cell survival in growth factor-depleted medium. However, we observed no features of morphological transformation, foci or the presence of viable cells after 2–3 weeks post-treatment (Figure 4B). Collectively, these results indicate that while certain normal cell types may take up and transiently retain the oncogenic cargo of tumor-derived EVs [8, 33], or exhibit biological responses to this material, these effects are transient in nature.

### H-*ras*-containing extracellular vesicles may compromise the viability of recipient cells

**Figure 5:**
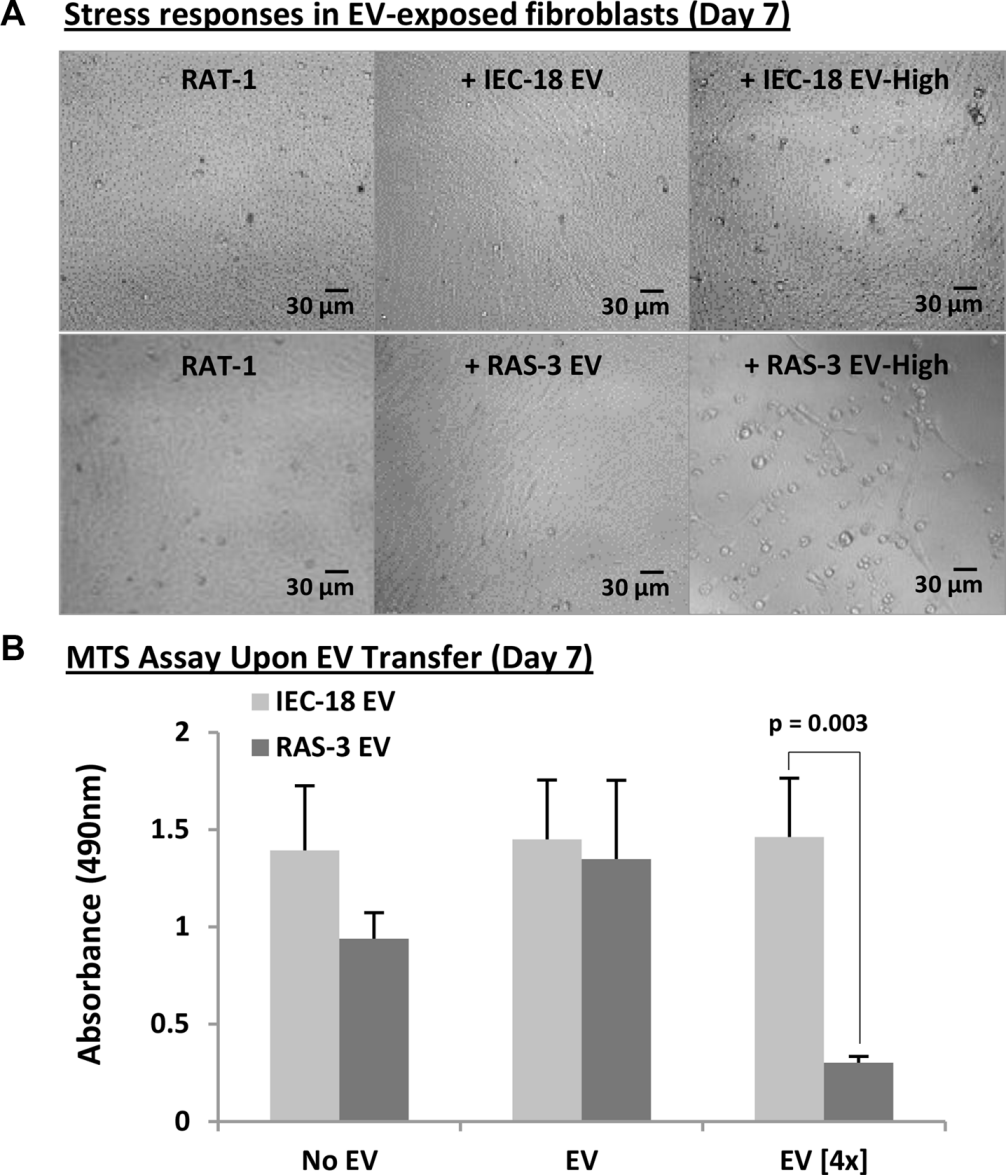
Elevated concentrations of H-*ras*-containing EVs compromise the survival of recipient fibroblasts (**A**) Morphological evidence (phase contrast microscopy) of cellular stress and death (detachment) of RAT-1 fibroblasts exposed for 7 days to concentrated (4×) EVs purified from RAS-3 conditioned medium. No such effect accompanied lower EV concentrations or concentrated EVs from non-tumorigenic IEC-18 cells. Images were taken on day 7 at 200x magnification. (**B**) MTS measurements of changes in metabolic activity of RAT-1 cells following treatment with indicated preparations suggest toxic effects of H-*ras*-containing EVs. Data are presented as the means ± SD and are representative of three independent experiments *P*-values as indicated.

We reasoned that escalated exposure to EVs containing mutant H-*ras* might increase the probability of horizontal transformation of recipient cells with a full-length DNA sequence [11]. Therefore, RAT-1 fibroblasts were cultured with either standard or quadrupled concentrations of EVs isolated from conditioned media of either RAS-3 cells or their non-transformed IEC- 18 counterparts (Figure 5). As expected, the latter preparations had no effect on the growth and viability of RAT-1 recipients. Interestingly, treatment with high concentrations of EVs from RAS-3 donors not only failed to facilitate horizontal transformation of RAT-1 fibroblasts, but instead resulted in cell rounding, detachment, decline in metabolic activity and cell viability, as measured by the MTS assay (Figure 5B). Thus, in this setting the increased exposure to cancer-derived EVs triggers cell death responses rather than horizontal transformation.

### Inability of H-*ras*-transformed cancer cells to trigger horizontal transformation *in vivo*

The ability of cancer cells to trigger malignant conversion of their normal counterparts *in vivo* is central to the notion of horizontal transformation [14, 18, 19]. To explore this possibility, we employed RAS-3 cells as extracellular H-*ras* donors under several experimental conditions. First, RAT-1 cells pre-treated for 24 hours with RAS-3-derived EVs were injected subcutaneously into immune-deficient SCID mice and their tumorigenic potential compared to that of untreated RAT-1 and RAS-3 controls (Table 1 and Supplementary Figure S6). In addition, RAT-1 or MEFp53−/− fibroblasts were co-injected into SCID mice in mixture with viable RAS-3 cells, whose mitogenic activity was arrested by pre-treatment with Mitomycin C (MitoC). In this case, the viable or dying RAS-3 cells would be expected to continually supply oncogenic H-*ras* into the immediate proximity of recipient RAT-1 cells (as EVs, ABs or free gDNA) [18]. Indeed, MitoC-treated RAS-3 cells labelled with Luciferase remained viable and detectable at the site of injection for up to 7 days post-inoculation, (Supplementary Figure S7A). Also, vesiculation by these cells remained unaffected by the MitoC treatment (Supplementary Figure S7B). Finally, MitoC-treated RAS-3 cells were injected alone, in which case their derived EVs (extracellular H-*ras*) could interact with resident mouse cells either at the site of injection, or systemically [35] (Table 1 and Supplementary Figure S7).

**Table 1:** The absence of permanent horizontal transformation (tumorigenic conversion) of normal cells exposed to oncogenic EVs and non-dividing cancer cells *in vivo*

Group	Tumors/Injection	Endpoints (Days)	Max Observation Time (Days)
RAS-3	6/6	12, 12, 12, 16, 16, 16	16
RAT-1	7/15	101, 129, 144, 163, 163, 176, 176	176
RAT-1 + RAS-3 EV	7/15	115, 158, 158, 158, 183, 192, 278	278
RAT-1 + MitoC_RAS-3	10/15	106, 119, 119, 119, 143, 150, 165, 169, 172, 192	192
MEFp53−/−	0/5	N/A	351
MEFp53−/−+ MitoC_RAS-3	0/7	N/A	351
MitoC_RAS-3	0/10	N/A	338

RAS-3 cells were used as donors of extracellular oncogenic activity, and the impact of this material on RAT-1 cells, MEFp53−/− or resident mouse cells was observed for up to 351 days (see Supplementary Figures S6–S8). No evidence of horizontal transformation was recorded.

We observed that while intact RAS-3 cells rapidly formed aggressive tumors in 100% of mice, which reached the endpoint within less than 3 weeks post-inoculation, this was not the case for other aforementioned experimental groups. As expected, control RAT-1 cells initially did not form tumors, but such outgrowths eventually emerged in 7 out of 15 injected mice after 101 to 176 days of latency. Surprisingly, the pre-treatment of RAT-1 cells with RAS-3-derived EVs or their co-injection with MitoC-treated RAS-3 cells did not significantly enhance their tumor-forming potential, as the lesions emerged in 7 and 10 out of 15 mice, respectively, and within 9 months of injection. Moreover, co-injection of MEFp53−/− cells with MitoC-treated RAS-3 cells resulted in no tumor formation, and neither did the injection of the latter cells alone (even after 338 days of observation). While the inability of MitoC-treated RAS-3 cells to form tumors is in agreement with published data [18], it effectively negates the ability of these aggressive cancer cells to transform adjacent normal host tissues in mice.

We also analyzed tumor samples for human H-*ras* sequences using PCR assays, including digital-droplet PCR (Supplementary Figure S8A and S8B). Of note, H-*ras* gDNA was only detectable in control RAS-3 tumors and was absent from tumors that emerged upon RAT-1 cell injection, either intact or following pre-treatment with RAS-3-derived EVs, or in mixture with RAS-3 cells treated with MitoC. In Southern blots, the H-*ras* hybridization bands following electrophoresis of gDNA restriction fragments were also distinctly different between RAS-3 and RAT-1 tumors, irrespectively of treatment (data not shown), as was the tumor morphology (Supplementary Figure S8C). Overall, these results are consistent with the notion that EV-mediated molecular transfer and the direct contact between RAS-3 donor cells and various susceptible cellular recipients fails to produce horizontal transformation *in vivo*.

## DISCUSSION

Our study suggests that while tumor-derived EVs containing oncogenic H-*ras* may trigger unique biological responses, some reminiscent of malignant transformation, these effects are restricted in scope and duration, and unable to trigger tumorigenic conversion of indolent cells. This is at variance with recent suggestions that extracellular oncogenes may activate a horizontal pathway of tumor progression whereby malignant features could be passed between clonally unrelated cellular populations [14, 17–19].

These observations are important, but not surprising, as clonal (rather than horizontal) evolution of cancer cells is supported by lineage tracing and phylogenetic analyses of cancer cell genomes in primary and metastatic cancers [23, 36, 37]. Moreover, the horizontal transformation model would imply a stochastic, polyclonal and multifocal progression of human malignancies with emergence of different tumor histotypes in the same patient, which is not a common clinical experience [38]. Normal cells would also be expected to undergo apoptotic death, or senescence (rather than transformation), upon transduction with potent extracellular oncogenes [31]. In keeping these predictions, we observed no permanent intercellular transfer of oncogenic gDNA, while other EV-associated transforming cargo, such as oncoproteins, mRNA or microRNA lack self-replicating potential, and their effects on cells could be inherently self-limiting due to degradation and dilution [6, 8]. Our study does not rule out the possibility of horizontal transformation mediated by oncogenic viruses, induction of genetic instability or epigenetic influences in the context of specific cancer cell types [22, 39, 40].

Using the well-defined source of oncogenic EVs containing mutant H-*ras* in all molecular forms (protein, mRNA and DNA) led us to the identification of several biological barriers that may curtail the extent of interactions between extracellular oncogenes and recipient cells. For example, while non-tumorigenic IEC-18 cells readily undergo malignant transformation upon experimental introduction of the H-*ras* oncogene, this effect cannot be recapitulated by H-*ras*-containing EVs, at least in part due to their poor cellular uptake. Interestingly, we observed that enforced or spontaneous malignant transformation triggers a dramatic increase in the uptake of exogenous EVs by indolent epithelial cells (IEC-18) and astrocytes (NHA) [6, 35, 41]. Our data suggest that these events occur downstream of activated RAS or SRC, but do not depend on MEK/MAPK (are not blocked by PD98059). Therefore, other RAS-regulated mechanisms remain of interest, including PI3K signalling, lipid metabolism, membrane dynamics and cytoskeleton [27].

We found that several types of indolent mesenchymal cells are susceptible to the EV uptake, including primary endothelial cells and fibroblastic cell lines of rat or mouse origin [11, 33]. These cells retained the exogenous H-*ras* gDNA and exhibited marked biological responses to EV treatment, including enhanced viability [11], production of VEGF (data not shown), phenotypic transformation and growth, as exemplified by formation of three-dimensional foci by immortalized fibroblasts. However, these changes were transient in nature, self-limiting and did not amount into permanent horizontal transformation, genomic integration or continued expression of the H-*ras* oncogene over long-term culture (see SI).

While horizontal transformation [14, 18, 19] and “genometastasis” [17] represent fascinating possibilities in the realm of EV-mediated intercellular communication, they were not observed in our experiments *in vivo*. Thus, co-injection of MitoC-treated RAS-3 cells with RAT-1 or MEFp53−/− fibroblasts, both susceptible to oncogenic transformation, or their pre-treatment with RAS-3-derived EVs did not increase the incidence of tumor formation above the background levels. Perhaps, the most startling observation in this regard is the fact that a protracted presence of viable, but non-dividing RAS-3 (MitoC-treated) cells at the injection site did not trigger tumor growth in surrounding mouse tissues. This is consistent with published observations where injection of MitoC-treated MDA-MB-231 breast cancer cells harboring mutant K-*ras* [18, 42] also failed to elicit malignant growth in adjacent normal tissues.

It is possible that horizontal transformation may require pre-existing alterations in recipient cells exposed to the uptake of EVs, ABs or DNA [14, 17, 19]. Arguably such permanent transforming influences cannot be presently excluded in specific contexts, such as cancer susceptibility syndromes (e.g. Li-Fraumeni, Lynch) characterized by wide-spread losses in tumor suppressor mechanisms, genetic instability and sub-threshold transformation-like states [43]. Whether horizontal transformation may occur in such settings remains to be documented.

Overall, we suggest that the possibility of *de novo* horizontal transformation of normal cells in association with sporadic cancers should be considered in the context of genetic [23], histological [38] and biological evidence, including the barrier mechanisms highlighted in our study. We propose that oncogenic EVs represent an important regulatory and communication mechanism, but not necessarily a pathway of permanent and genetic horizontal transformation. We also suggest that EV trafficking may favour already transformed and mesenchymal cells over their indolent and epithelial counterparts.

## MATERIALS AND METHODS

### Cell lines and culture conditions

The following rat cell lines were used in the study: IEC-18 – non-tumorigenic, immortalized rat intestinal epithelial cell line; RAS-3 – tumorigenic, clonal IEC-18 subline that has been transfected with a V12 mutant, activated c-H-*ras* human oncogene; SRC-3 – tumorigenic, clonal variant of IEC-18 cells that has been transfected with the v-*src* oncogene; RAT-1 – non-tumorigenic, immortalized rat fibroblast cell line; RAT-2 – non-tumorigenic; immortalized rat fibroblast cell line. The IEC-18, RAS-3 and SRC-3 cells were grown as previously described [34]. The RAT-1 and RAT-2 cells were maintained in DMEM supplemented with 10% FBS and 1% Pen/Strep. The following human cell lines were used in the study: NHA – normal human astrocyte cell line immortalized using human telomerase (*HTERT*); U373 – parental non-tumorigenic/indolent human glioblastoma cell line; U87 – parental tumorigenic glioma cell line; DAOY – human medulloblastoma cell line; HUVEC – normal, non-tumorigenic human umbilical vein endothelial cells were purchased from the ATCC (#CRL-2873). The U373 and U87 cells were gifts from Dr. Abhijit Guha, University of Toronto. The DAOY cells were a gift from Dr. Nada Jabado, McGill University). The NHA, U373 and U87 cells were maintained in DMEM supplemented with 10% FBS (#080–150, Wisent Bioproducts) and 1% Pen/Strep (#15140, GIBCO). Monolayer cultures of DAOY cells were cultured in DMEM/F12 supplemented with 10% FBS [44]. HUVEC cells were maintained according to the manufacturer's protocol. The NHA Low Passage (L), Intermediate Passage (I) and High Passage (H) designations refer to NHA cells that were passaged for 1–10, 11–20 and 21–30 subculture cycles at the ratio of 1:10, respectively. The NIH3T3 mouse embryonal fibroblasts were cultured in DMEM supplemented with 10% FBS. P53-deficient mouse embryonic fibroblasts (MEFp53−/−) were maintained in DMEM supplemented with 10% FBS and 1% Pen/Strep. For experiments involving EV isolation, the media were supplemented with 10% EV-depleted FBS (ultracentrifugation at 150,000-g for 5 hours). Detailed information can be found in the Supplementary Appendix.

### Soft agar colony formation assay

Unless otherwise indicated 5,000 respective cells were grown in soft agar (base: 1% agar; top: 0.7% agar) for 2–4 weeks. Fresh media was added every 5–7 days. The multicellular colonies were counted using the inverted microscope at 100× magnification. The field was divided into 4 quadrants, and the total number of colonies was determined by adding up the values. The average number of colonies was calculated based on 3 independent repeats.

### Isolation of EVs and their biological assays

EVs were obtained by ultracentrifugation as previously described [6, 11, 33, 45]. The resulting pellet was resuspended in complete media and incubated overnight (PKH26 transfer assay) or for several days (foci formation assay) with recipient cells (see below and Supplementary Appendix).

### Transmission electron microscopy (TEM)

Cells and isolated EVs were washed once with 0.1 M sodium cacodylate buffer (pH 7.4) and fixed with 2.5% glutaraldehyde in the same buffer. The fixed samples were then subjected to either ultramicrotomy for processing of monolayer cells or whole mount negative staining for capturing of all EVs in the fixative. The Tecnai 12 BioTwin 120 kV TEM was used to capture images.

### Tracking of cellular EV uptake through the use of PKH26 fluorescence

RAS-3 cells were labelled with PKH26 (#MINI26, Sigma-Aldrich) red fluorescent dye as described [11]. Briefly, a single cell suspension of RAS-3 cells was washed once with serum-free DMEM, after which PKH26 was added for 5 minutes at room temperature with periodic mixing. The staining was stopped by adding an equal volume of FBS for 1 minute and then an equal volume of complete medium. The labelled cells were washed 3 times with complete media and subsequently cultured until 80–90% confluent. EVs obtained from these cultures were resuspended in complete media and incubated overnight with 1.5 × 10^5^ recipient cells (IEC-18, SRC-3, RAS-3, NHA (L), NHA (I), NHA (H), RAT-1, RAT-2, NIH3T3, MEFp53−/−, U87, U373 and DAOY cells), which were analyzed by FACS for the PKH26 fluorescence transfer.

### Foci formation assay

EVs obtained from 4 cell culture dishes (100 mm) of RAS-3 cells by ultracentrifugation were resuspended in complete media and incubated overnight with 1.5 × 10^5^ RAT-1 cells, which were serum-starved for approximately 6 hours prior to the EV treatment. The recipient cells were trypsinized the following day and then re-plated into four 35 mm cell culture dishes for their long-term culture. Media was changed every 7 days, and the colonies were counted every 5 days using the inverted microscope. Images of cells/foci were taken at 40x, 100x and 200x magnifications. Four foci were picked and expanded from the EV-treated, foci-forming RAT-1 cell cultures on day 36 post plating (clones #1, #2, #3, #4 and RAT-1 as control). This was to assess whether their colony-forming ability is retained or lost during an even longer period in culture. These cells were passaged, upon confluence, for up to 6 cycles (80–100 days) until the complete loss of their foci-forming potential was noticeable.

### EV transfer assay

Primary cultures of human umbilical vein endothelial cells (HUVEC) were used as recipients for RAS-3- or IEC-18-derived EVs, as controls. Briefly: 5,000 HUVEC cells were first cultured in the presence of EVs under growth factor starvation conditions (DMEM supplemented with 1% FBS) for 10 days and complete EBM2 media was added afterwards. Initially, the effect of growth factors present in EV cargo was assessed by counting the number of attached (i.e. viable) cells under starving conditions. Next, the cells were no longer starved and complete EBM2 media was added once every week to observe resumption of cellular growth and formation of transformed foci (if any). Inverted microscope images of recipient cells were taken regularly at 100x and 200x magnifications.

### Measurements of recipient cell viability

EVs obtained at standard or quadruple concentrations from RAS-3 and IEC-18 cells by ultracentrifugation were resuspended in complete media and incubated with 4,500 RAT-1 cells. These cells were plated in 96 well plates and analyzed for the MTS (#G3580, Promega) signal after 7 days. Microscope images of RAT-1 cells were taken regularly at 200x magnifications.

### Tumor formation assays

The indicated numbers of cells were injected subcutaneously into severe combined immunodeficient mice (SCID) as indicated (see Supplementary Appendix). Tumor growth was followed by palpation and caliper measurements, and tissues were obtained at autopsy for histological and molecular analysis. Animal material was obtained according to the protocol approved by the Facility Animal Care Committee at our Institution and in agreement with Guidelines of the Canadian Council of Animal Care.

### Data analysis

All experiments were performed at least twice with similar results. Numerical data were processed for significance using two-tailed *t*-test with the threshold *p*-value of 0.05.

## SUPPLEMENTARY MATERIALS FIGURES


