# Supplementary material for: Barriers to horizontal cell transformation by extracellular vesicles containing oncogenic H-ras
Source: Oncotarget. 2016 Jul 16;7(32):51991–2002. doi: 10.18632/oncotarget.10627 (PMC5239530; doi:10.18632/oncotarget.10627)
Supplement: Supplementary file 1 [file oncotarget-07-51991-s001.pdf]

Barriers to horizontal cell transformation by extracellular vesicles containing oncogenic H-*ras*

Supplementary Materials

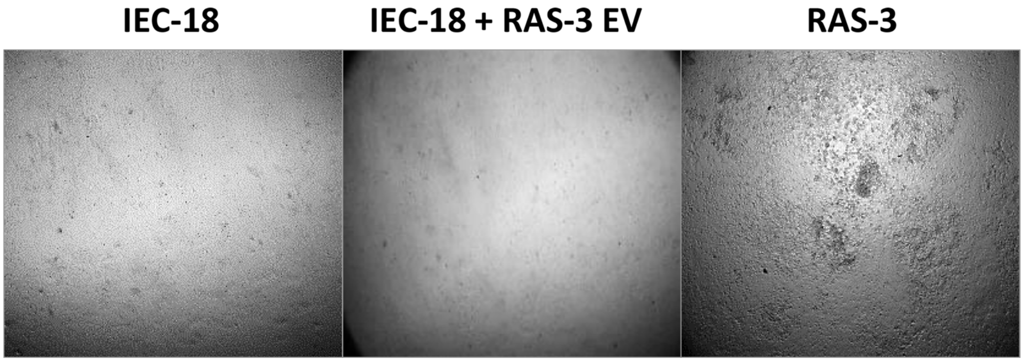

Supplementary Figure S1: Failure of RAS-3-derived EVs to induce foci formation in cultures of parental IEC-18 cells. Untreated IEC-18 cells exhibit a differentiated cobblestone morphology and form flat monolayer culture surfaces that undergo contact inhibition upon confluence. In contrast, their aggressive H-*ras* transfectants (RAS-3) are spindle-shaped and spontaneously form dome-like and spherical structures upon reaching high density. Addition of RAS-3-derived EVs to IEC-18 cultures does not alter their growth patterns. Images were taken at 40x magnification.

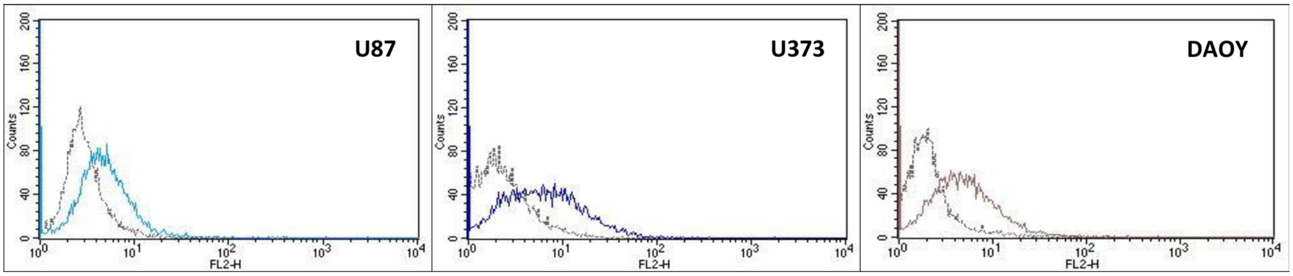

Supplementary Figure S2: Efficient uptake of RAS-3-derived fluorescent EVs by brain cancer cells. As shown in Fig. 2, the non-transformed, differentiated epithelial and astrocytic cells are refractory to EV uptake, while this property is reversed by malignant transformation. Therefore, we tested several cancer cell lines isolated from human glioma (U373, U87) or medulloblastoma (DAOY) using standardized uptake assays involving PKH26-labelled EVs purified from RAS-3 cell cultures. All cancer cell lines tested were able to avidly take up fluorescent EVs, as indicated by the shift in FL2 profile (coloured lines) in comparison to untreated controls (grey lines) in FACS histograms.

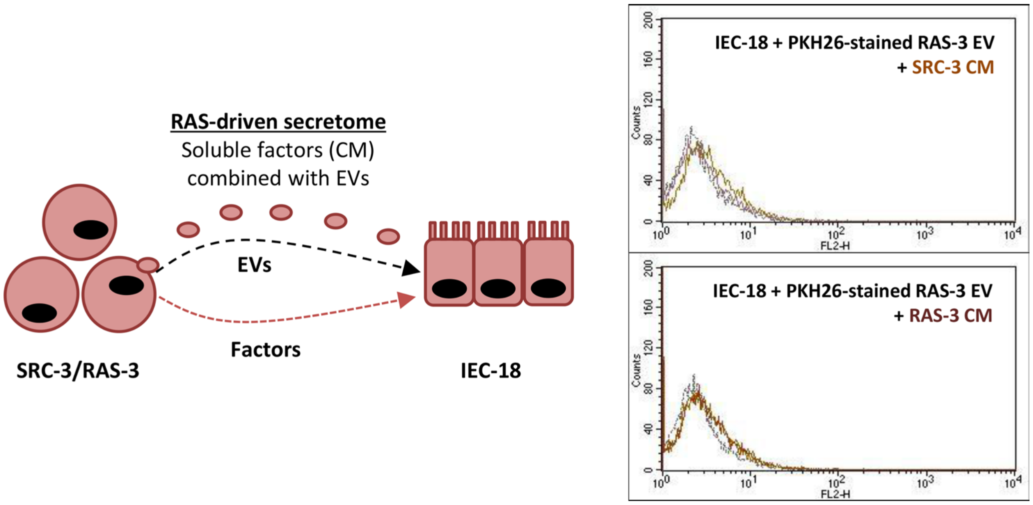

Supplementary Figure S3: Secreted factors from RAS-3 cancer cells are unable to recapitulate the effects of endogenous mutant H-*ras* expression on the EV uptake by non-tumorigenic IEC-18 cells. H-*ras* transformation changes cellular secretome, thereby contributing to the expression of malignant phenotype. We asked whether these paracrine secreted factors (e.g. growth factors, enzymes) may regulate EV uptake in IEC-18 cells similarly to the effects exerted by the endogenous mutant H-*ras*. To accomplish this, RAS-3 EVs labelled with PKH26 were added to IEC-18 cultures in the presence or absence of the soluble fraction of RAS-3 conditioned medium (CM). FACS analysis revealed that this treatment did not change EV uptake, and thereby did not recapitulate the effects of endogenous H-*ras* expression (See Figure 2).

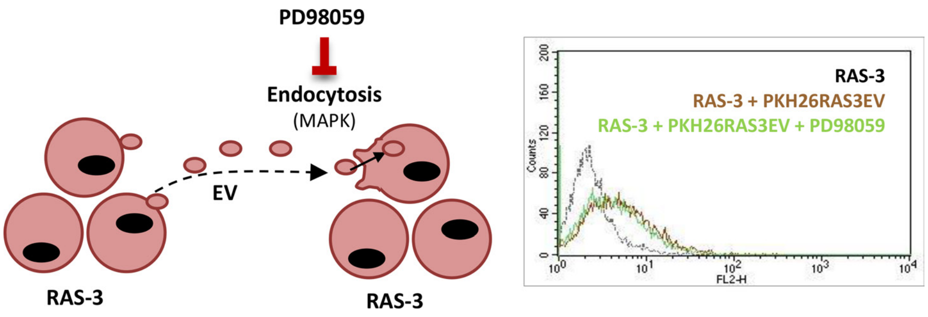

Supplementary Figure S4: MEK/MAPK pathway activity is not required for EV uptake by H-*ras*-transformed cancer cells. Enforced HRAS transformation in IEC-18 cells (RAS-3 clone) leads to the onset of EV uptake. Since HRAS activates the MAPK cascade, which has been implicated in EV endocytosis, we asked whether blockade of the MEK/MAPK pathway using the pharmacological inhibitor PD98059 leads to the reversal of HRAS-mediated EV uptake (See Figure 2). RAS-3 cells were pre-treated for 24 hours with PD98059 at 50 μM or with vehicle and subsequently exposed to PKH26-labelled EVs purified from RAS-3 cell cultures. No change in the avid transfer of EV fluorescence was observed under these conditions, suggesting that MEK/MAPK activity is not essential for oncogene-induced uptake of EVs.

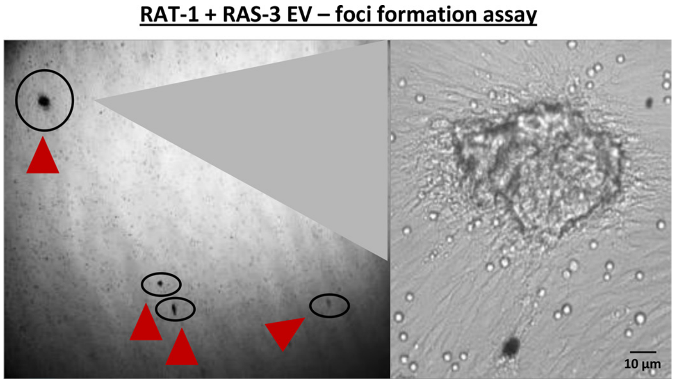

Supplementary Figure S5: Formation of transformed foci by RAT-1 fibroblasts treated with EVs containing mutant H-*ras*. We observed that cultured RAT-1 fibroblasts readily take up EVs purified from RAS-3 cancer cells harboring mutant H-*ras* oncogene. Following this treatment, RAT-1 cells begin to form dome-like foci consisting of phenotypically transformed cells pilling up above monolayers (arrowheads, enlargement of the RAT-1 focus – right side panel, see Figure 2). Images were taken on day 10 at 40x (left) and 200x (right) magnifications.

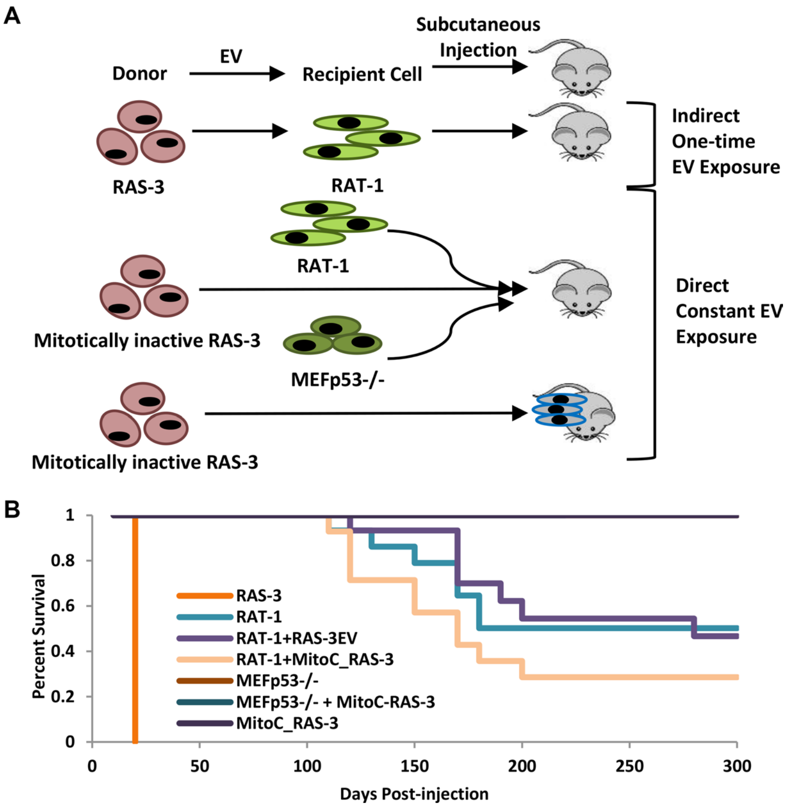

Supplementary Figure S6: Studies interrogating the ability of extracellular oncogenic activity associated with RAS-3 cells to act as a potential trigger of horizontal transformation *in vivo*. (**A**) Experimental design: Immune-deficient SCID mice were used as recipients of subcutaneous grafts consisting of 2 × 10^6 viable RAT-1 cells pre-treated in culture with RAS-3-derived EVs, as in Figure 2. Alternatively, RAT-1 cells were injected in mixture with mitotically inactive but viable RAS-3 cells (5 × 10^4), which had been pre-treated with Mitomycin C (MitoC). In a similar manner, injections of MEFp53–/– primary fibroblasts were carried out. Finally, Mitomycin C-treated RAS-3 cells were injected alone, in which case the recipients of their related extracellular transforming activity would be only the host (mouse) cells at the site of injection or systemically. Untreated RAS-3, RAT-1 and MEFp53-/- cells were injected as controls. (**B**) Cumulative survival of mice upon injection of the aforementioned cellular preparations (Kaplan-Meier curves). All mice injected with RAS-3 cells rapidly developed aggressive tumors, while spontaneous growth of RAT-1 cells occurred in a fraction of mice and after prolonged latency. No increase in tumor formation due to horizontal transformation or intercellular transfer of oncogenic activity from RAS-3 cells to non-transformed recipients was observed in these experiments (see Table 1).

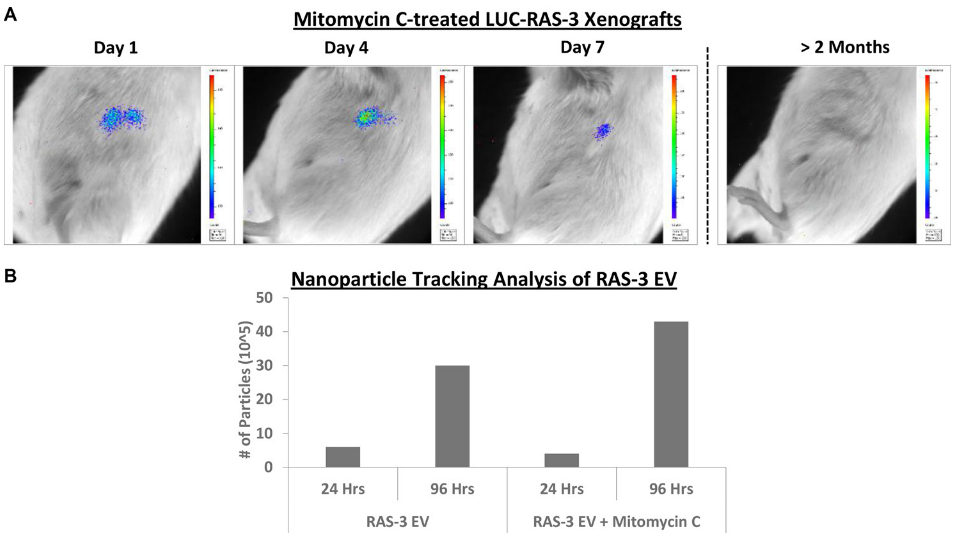

Supplementary Figure S7: Retention of viable Mitomycin C-treated RAS-3 cells at the injection site. (**A**) RAS-3 cells expressing Luciferase were generated and 5 × 10^4 cells were injected subcutaneously into SCID mice (see Figure 4). Monitoring bioluminescence revealed the presence of viable cancer cells in numbers exceeding detection threshold for up to 7 days. The signal eventually decayed, suggesting the removal of these cells from tissue. (**B**) Mitomycin-treated RAS-3 cells continue producing EVs. Conditioned media of RAS-3 cells were collected in the absence and presence of Mitomycin C to obtain their total number of nanoparticles emitted. The analyses were performed by NTA.

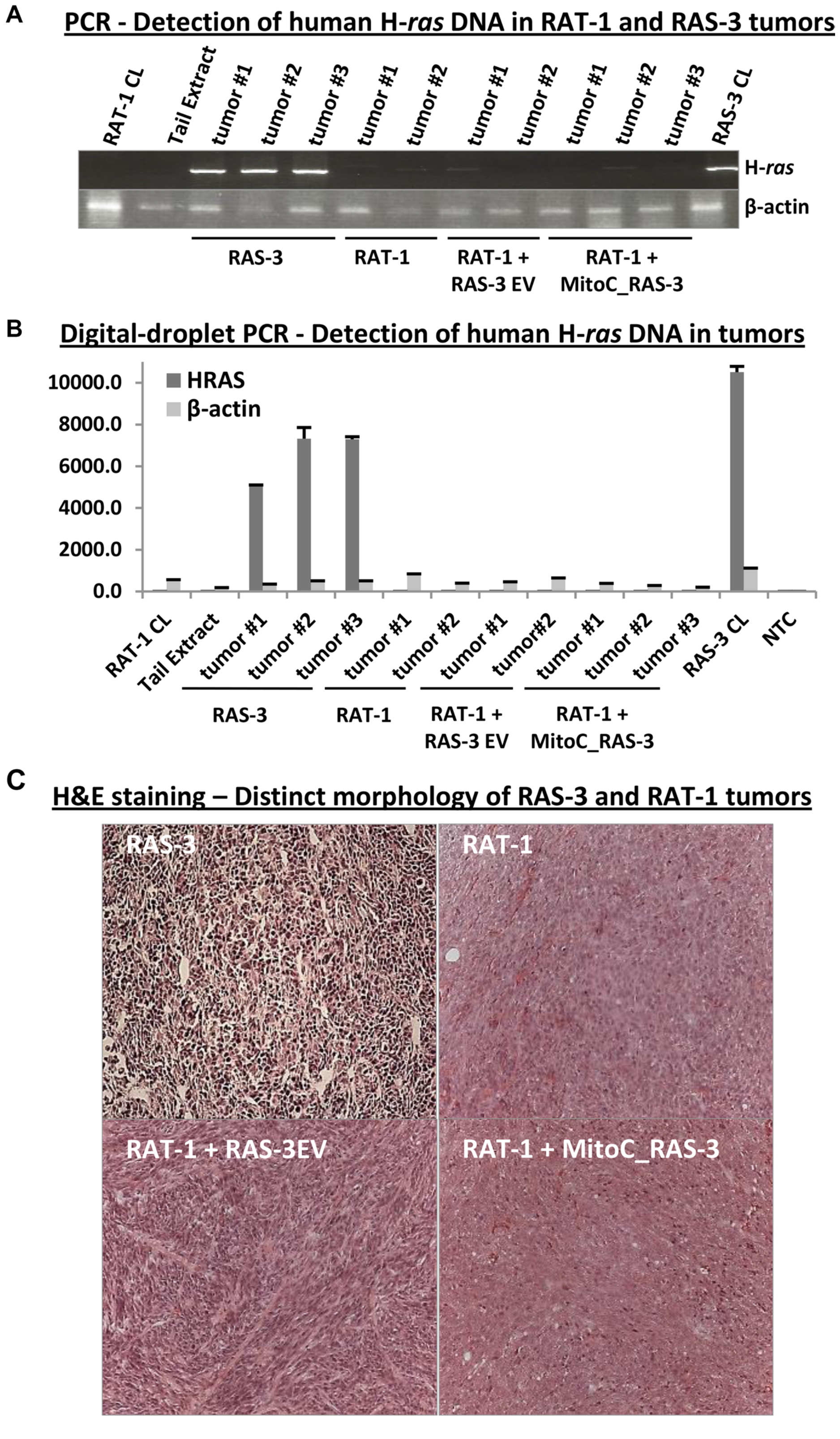

Supplementary Figure S8: Mutant H-*ras* gDNA transfer does not contribute to tumorigenicity of RAT-1 fibroblasts in mice. No human H-*ras* genomic sequences were detected in tumors induced by RAT-1 cell injections, regardless of prior exposure of these cells to RAS-3 cells or their EVs. (**A**) PCR analysis of human-specific genomic sequences corresponding to mutant H-*ras* oncogene in indicated tumors. RAS-3 cells and xenografts harboring the human H-*ras* transgene are positive for this signal, while none of the tumors resulting from injection of RAT-1 cells, either in mixture with RAS-3 cells or upon treatment with RAS-3-derived EVs, exhibits signs of human H-*ras* presence. (**B**) Digital-droplet PCR analysis showing the presence of human H-*ras* oncogene only in xenografts containing RAS-3 cells. C, RAS-3 and RAT-1 tumors exhibit different histology upon H&E staining.

MATERIALS AND METHODS

Detailed description of materials, primers and reagents

The following antibodies were used throughout the study: Ras (27H5; #3339, Cell Signaling) and Flotillin-1 (#610821, BD Biosciences). The following primers were used throughout the study: H-*ras* mRNA (forward 5′ – TCAAACGGGTGAAGGACTCG), H-*ras* mRNA (reverse 5′ – CTGCGTCAGGAGAGCACAC), human-specific H-*ras* #1 (forward 5′ – GCAGGAGACCCT GTAGGAGGACCC), human-specific H-*ras* #1 (reverse 5′ – TGGCACCTGGACGGCGGCGCCAG), human-specific H-*ras* #2 (forward 5′ – TCCCTTTAGCCTTTCTGCCG), human-specific H-*ras* #2 (reverse 5′ – CCCATCAATGA CCACCTGCT), GAPDH (forward 5′ – TGCACCAC CAACTGCTTAGC), GAPDH (reverse 5′ – GGCAT GGACTGTGGTCATGAG), β-actin (forward 5′ – GCACC ACACCTTCTACAATGA) and β-actin (reverse 5′ – TCA TCTTCTCGCGGTTGGC). PCR amplifications of the H-*ras* oncogene were done using human-specific primers unless otherwise indicated. Inhibition of the MEK/MAPK pathway in cells was achieved by treating them with 50 µM PD98059 (#513000, Calbiochem). Inhibition of the Na+/H+ exchanger (NHE) in cells was achieved by treating them with 75 µM ethyl-isopropyl amiloride (EIPA) compound (#A3085, Sigma).

Immunofluorescence

Cultured cells were fixed for 10 minutes in 4% paraformaldehyde, permeabilized with 0.2% Triton X-100 and stained overnight at 4°C with antibodies. This was followed by their respective secondary Alexa Fluor antibodies (Invitrogen). Imaris software (Bitplane) was used for the analysis of confocal images.

Detailed EV isolation methods

EVs were obtained at standard (2 100 mm petri dishes, each containing 9 ml of conditioned media and 0.65 µg/µl of EV protein) or quadruple concentrations by ultracentrifugation as previously described [1–5]. Briefly, conditioned media was centrifuged at 400-g for 10 minutes to remove cell debris, followed by filtration through a 0.2 µm PES filter (#565–0020, Thermo Fisher Scientific). The filtrate was ultracentrifuged at 110,000-g for 1 hour to isolate EVs. For sucrose gradient centrifugation [5], the filtrate was centrifuged at 110,000-g for 1 hour, and the pellets were resuspended in 2 ml of 20 mM HEPES, 2 M sucrose. The resulting samples were transferred to the bottom of the Beckman SW41 centrifuge tube and slowly layered with a continuous gradient of sucrose, from 2 to 0.25 M. The samples were ultracentrifuged in a swinging bucket rotor for 17 hours at 210,000-g with the brake set on low. After centrifugation, the samples in each tube were separated into 10 fractions of 1 ml each, starting from the top. Each fraction was transferred into a 3-ml tube, and mixed with 2 ml of HEPES (20 mM), centrifuged at 110,000-g for 1 hour, resuspended in 50 µl of PBS. EV protein extracts were obtained by directly adding radioimmunoprecipitation assay buffer (RIPA) to the pellet after the spin. Similarly, EV nucleic acid extracts were obtained by either adding Trizol (RNA) or proteinase K-treated lysis buffer (DNA). For the total number of particles, conditioned media were loaded onto the nanoparticle tracking analysis system (NTA; #NS500, Nanosight) and five recordings of 30 seconds were obtained and processed using NTA software.

Detailed digital droplet PCR method

The ddPCR reaction mix consists 1x EvaGreen ddPCR Supermix (#1864034, Bio-Rad), 100 nM H-*ras* or ß-actin primers and 2 µl of the DNA sample. Over 15,000 droplets were generated using 60 µl of Droplet Generation Oil (Bio-Rad) and 20 µl of PCR reaction mix using QX200 Droplet Generator (#1864005, Bio-Rad). These droplets are carefully transferred to 96-well plates and sealed. PCR was performed in a C1000 Touch Thermal Cycler (Bio-Rad) with the following cycling conditions for H-*ras*: 1 × (95°C for 5 minutes), 45 × (95°C for 30 seconds, 64°C for 30 seconds, 72°C for 30 seconds), 1 × (4°C for 5 minutes, 90°C for 5 minutes) and ß-actin: 1 × (95°C for 5 minutes), 45 × (95°C for 30 seconds, 57°C for 30 seconds, 72°C for 30 seconds), 1× (4°C for 5 minutes, 90°C for 5 minutes). Finally, the fluorescence intensity of the amplified droplets was analyzed using the QX200 Droplet Reader (Bio-Rad).

Detailed histology (H&E staining)

Tumor samples from xenograft experiments were fixed in 4% paraformaldehyde immediately upon their resection from the mice. A series of automated processing steps were then executed on the samples by the Leica TP 1050 tissue processor. The resulting paraffin-embedded blocks were sectioned into 4 µm thick tissue sections using the American Optical microtome and were placed on pre-coated microscope slides. These slides were de-waxed in xylene and then hydrated in a series of washes from 95% ethanol to 50% ethanol. The slides were visualized by haematoxylin and eosin (H&E) staining, which was done by incubating them with 1.5% haematoxylin, pH 2.5, Blueing solution and eosin solution.

Supplemental discussion and references

Intercellular transfer of molecular information constitutes the key element of cancer complexity and defines the systemic impact of many human cancers, whether localized or metastatic [6]. These processes are not stochastic or 'unspecific' but rather represent the extracellular extension of malignant transformation, which is now known to encompass angiogenesis [7],

coagulopathy [8–10], as well as inflammatory, immune and stromal effects [11–13], and metastasis [14–22]. Indeed, RAS and other oncogenic pathways may be responsible for modulating secretomes and phenotypes of adjacent normal cells through soluble, adhesive and other ‘field’ effects [23]. In this regard the intercellular trafficking of cancer cell-derived EVs has been implicated in several biological responses, including modulation of cell growth, survival, drug resistance, immunoregulation, stemness, angiogenesis and metastasis [2, 12, 14, 15, 24–26].

There is mounting evidence to suggest that cancer-related EVs contain and mediate intercellular transfer of potent oncogenic, signalling and regulatory molecules, including proteins, microRNA, mRNA and DNA [25, 27–35]. The latter include epidermal growth factor receptor (EGFR/EGFRvIII), [2, 4, 26], HER-2 [36], MET [15], KIT [37], AKT [38], BCR-ABL [34], LMP1 [39] mutant K-*ras* [40], H-*ras* [3], and possibly several others[6]. Cancer-related EVs may also contain bioactive tumor suppressors [41, 42], regulatory proteins [43], microRNA [44], mRNA [24, 26, 45] transposons, single-stranded DNA [28], as well as histones, chromatin and gDNA [3, 29, 30]. The intercellular transfer of these entities demonstrably changes the functional state and phenotype of recipient cells, of which cancer cell subpopulations, endothelium, myeloid cells, normal epithelium and stroma constitute the most studied examples [4, 15, 26, 46].

In our study, EVs released from RAS-3 cells exerted pro-survival effects in the context of primary human endothelial cells. This is of interest as processes of tumor angiogenesis are often dissimilar from those occurring during normal vascular development, and may include changes in endothelial cells that resemble genetic transformation [47]. For example, tumor-related endothelial cells may exhibit aneuploidy [48], or oncogenic features of their adjacent cancer cells [49, 50]. The impact of oncogenic EVs on these events is uncertain but cultured endothelial cells exposed to EV-associated oncogenic EGFR acquire the ability to activate vascular endothelial growth factor receptor 2 (VEGFR2) in an apparently autocrine manner [4]. In this sense, cancer-related EVs may be viewed as unique mediators of abnormal tumor angiogenesis [6].

In our experiments, the effects of H-*ras*-containing EVs on HUVEC cells were consistent with their postulated role in tumor angiogenesis [4, 26]. This observation is in line with the mounting evidence for EV-mediated cell-cell communication, including intercellular transfer of potent oncogenic, signal-transducing and regulatory molecules to the vascular and other cellular compartments, as reviewed in [6].

As mentioned earlier, EV cargo may contain bioactive tumor suppressors [41, 42], bioactive regulatory proteins [43], microRNA [44], mRNA [24, 26, 45], single-stranded DNA [28], as well as histones, chromatin and gDNA [3, 29, 30]. It is of note that our study documents that mutant H-*ras* induces emission of genomic DNA as cargo of cancer-related EVs. The modulatory role of these molecular entities in cancer progression and metastasis is increasingly well documented *in vitro* and *in vivo* [4, 15, 16, 26]. Interestingly, we observed no obvious effect of EVs purified from IEC-18 conditioned medium.

Studies are underway to elucidate pathways governing EV uptake by various cell types, including the role of endocytosis mediated by proteoglycan and other mechanisms [51, 52]. The regulation of the intracellular fate and processing of different EV-related macromolecules by various types of recipient cells is of considerable interest but remains largely unclear. Published reports suggest cell-specific intracellular degradation [53], re-expression [2] or secretion [22] of cargo molecules, but the fate of EV-related DNA remains to be determined. It is possible that elimination of this material may involve removal of cells harboring EV-related DNA, as suggested by detrimental effects of the excessive EV uptake [54].

Finally, while we used transfer of H-*ras* gDNA to document molecular transfer between donor RAS-3 cells and different EV recipients, it is likely that multiple molecular perturbations downstream of RAS and secondary to H-*ras* transformation (e.g. due to genetic instability and drift) may influence the content and biological activity of RAS-3-derived EVs. For example, several RAS-regulated target and effector proteins, transcripts and microRNA and other non-coding RNA may contribute to changes we observed following the RAS-3 EV uptake [40]. Implicitly, some of these macromolecules may be essential for H-*ras*-dependent transformation and undergo intercellular transfer as cargo of EVs. These considerations notwithstanding, permanent transformation was not observed in any of the RAS-3-EV recipient cells analysed in our study. Thus, in cases where horizontal transformation occurs experimentally or *in vivo* upon contact of cancer and stromal cells is probably relatively rare and may entail viral transmission, epigenetic reprogramming or other secondary changes rather than direct transfer and reactivation of genomic sequences carrying mutant oncogenes. It is also possible that cellular responses to exogenous DNA may activate pathways of sequestration, elimination and stress response reminiscent of those triggered by viral infection. Indeed, we observed toxicity of EVs containing genomic DNA and derived from cancer cells, when added at high concentrations, but this was not the case for comparable levels of EVs derived from non-transformed cells and devoid of DNA. Mutant RAS represents one of the most potent transforming influences and a paradigm of molecular genesis of human cancers. While intrinsic barriers block these effects by provoking demise of normal cells exposed to mutant RAS signalling, so too, as shown in our study, do various normal cells populations mount biological barriers against transformation through horizontal transfer of RAS as cargo of extracellular vesicles.

REFERENCES

1. Yu JL, Rak JW. Shedding of tissue factor (TF)-containing microparticles rather than alternatively spliced TF is the main source of TF activity released from human cancer cells. J Thromb Haemost. 2004; 2:2065–2067.
2. Al-Nedawi K, Meehan B, Micallef J, Lhotak V, May L, Guha A, Rak J. Intercellular transfer of the oncogenic receptor EGFRvIII by microvesicles derived from tumour cells. Nat Cell Biol. 2008; 10:619–624.
3. Lee TH, Chennakrishnaiah S, Audemard E, Montermini L, Meehan B, Rak J. Oncogenic ras-driven cancer cell vesiculation leads to emission of double-stranded DNA capable of interacting with target cells. Biochem Biophys Res Commun. 2014; 451:295–301.
4. Al-Nedawi K, Meehan B, Kerbel RS, Allison AC, Rak J. Endothelial expression of autocrine VEGF upon the uptake of tumor-derived microvesicles containing oncogenic EGFR. Proc Natl Acad Sci U S A. 2009; 106:3794–3799.
5. Thery C, Clayton A, Amigorena S, Raposo G. Isolation and characterization of exosomes from cell culture supernatants and biological fluids; in: Current Protocols in Cell Biology, John Wiley & Sons, Inc., 2006, p. 3.22.1–3.22.29.
6. Rak J. Extracellular vesicles-biomarkers and effectors of the cellular interactome in cancer. Front Pharmacol. 2013; 4:21. doi: 10.3389/fphar.2013.00021. Epub;2013 Mar 6.:21.
7. Rak J. Ras oncogenes and tumour vascular interface; in: A.Thomas-Tikhonenko (ed): Cancer Genome and Tumor Microenvironment. New York, Springer, 2009, p. 133–65.
8. Yu JL, May L, Lhotak V, Shahrzad S, Shirasawa S, Weitz JI, Coomber BL, Mackman N, Rak JW. Oncogenic events regulate tissue factor expression in colorectal cancer cells: implications for tumor progression and angiogenesis. Blood. 2005; 105:1734–1741.
9. Milsom CC, Yu JL, Mackman N, Micallef J, Anderson GM, Guha A, Rak JW. Tissue factor regulation by epidermal growth factor receptor and epithelial-to-mesenchymal transitions: effect on tumor initiation and angiogenesis. Cancer Res. 2008; 68:10068–10076.
10. Magnus N, Garnier D, Rak J. Oncogenic epidermal growth factor receptor up-regulates multiple elements of the tissue factor signaling pathway in human glioma cells. Blood. 2010; 116:815–818.
11. Sparmann A, Bar-Sagi D. Ras-induced interleukin-8 expression plays a critical role in tumor growth and angiogenesis. Cancer Cell. 2004; 6:447–458.
12. Wolfers J, Lozier A, Raposo G, Regnault A, Thery C, Masurier C, Flament C, Pouzieux S, Faure F, Tursz T, Angevin E, Amigorena S, Zitvogel L. Tumor-derived exosomes are a source of shared tumor rejection antigens for CTL cross-priming. Nat Med. 2001; 7:297–303.
13. Boyango I, Barash U, Naroditsky I, Li JP, Hammond E, Ilan N, Vlodavsky I. Heparanase cooperates with Ras to drive breast and skin tumorigenesis. Cancer Res 2014; 74:4504–4514.
14. Hood JL, San RS, Wickline SA: Exosomes released by melanoma cells prepare sentinel lymph nodes for tumor metastasis. Cancer Res 2011; 71:3792–3801.
15. Peinado H, Aleckovic M, Lavotshkin S, Matei I, Costa-Silva B, Moreno-Bueno G, Hergueta-Redondo M, Williams C, Garcia-Santos G, Ghajar CM, Nitadori-Hoshino A, Hoffman C, Badal K, et al. Melanoma exosomes educate bone marrow progenitor cells toward a pro-metastatic phenotype through MET. Nat Med 2012; 18:833–891.
16. Hoshino A, Costa-Silva B, Shen TL, Rodrigues G, Hashimoto A, Tesic MM, Molina H, Kohsaka S, Di GA, Ceder S, Singh S, Williams C, Soplop N, et al. Tumour exosome integrins determine organotropic metastasis. Nature 2015; 19;527:329–335.
17. Poste G, Nicolson GL: Arrest and metastasis of blood-borne tumor cells are modified by fusion of plasma membrane vesicles from highly metastatic cells. Proc Natl Acad Sci U S A. 1980; 77:399–403.
18. Costa-Silva B, Aiello NM, Ocean AJ, Singh S, Zhang H, Thakur BK, Becker A, Hoshino A, Mark MT, Molina H, Xiang J, Zhang T, Theilen TM, et al. Pancreatic cancer exosomes initiate pre-metastatic niche formation in the liver. Nat Cell Biol. 2015; 17:816–826.
19. Jung T, Castellana D, Klingbeil P, Cuesta H, I, Vitacolonna M, Orlicky DJ, Roffler SR, Brodt P, Zoller M. CD44v6 dependence of premetastatic niche preparation by exosomes. Neoplasia. 2009; 11:1093–1105.
20. Thuma F, Zoller M: Outsmart tumor exosomes to steal the cancer initiating cell its niche. Semin Cancer Biol 2014;:10.
21. Gesierich S, Berezovskiy I, Ryschich E, Zoller M: Systemic induction of the angiogenesis switch by the tetraspanin D6.1A/CO-029. Cancer Res. 2006; 66:7083–7094.
22. Luga V, Zhang L, Viloria-Petit AM, Ogunjimi AA, Inanlou MR, Chiu E, Buchanan M, Hosein AN, Basik M, Wrana JL. Exosomes Mediate Stromal Mobilization of Autocrine Wnt-PCP Signaling in Breast Cancer Cell Migration. Cell 2012; 151:1542–1556.
23. Kalas W, Yu JL, Milsom C, Rosenfeld J, Benezra R, Bornstein P, Rak J. Oncogenes and Angiogenesis: down-regulation of thrombospondin-1 in normal fibroblasts exposed to factors from cancer cells harboring mutant ras. Cancer Res. 2005; 65:8878–8886.
24. Ratajczak J, Miekus K, Kucia M, Zhang J, Reca R, Dvorak P, Ratajczak MZ. Embryonic stem cell-derived microvesicles reprogram hematopoietic progenitors: evidence for horizontal transfer of mRNA and protein delivery. Leukemia. 2006; 20:847–856.
25. Valadi H, Ekstrom K, Bossios A, Sjostrand M, Lee JJ, Lotvall JO. Exosome-mediated transfer of mRNAs and microRNAs is a novel mechanism of genetic exchange between cells. Nat Cell Biol. 2007; 9:654–659.
26. Skog J, Wurdinger T, van RS, Meijer DH, Gainche L, Curry WT Jr, Carter BS, Krichevsky AM, Breakefield XO. Glioblastoma microvesicles transport RNA and proteins that promote tumour growth and provide diagnostic biomarkers. Nat Cell Biol. 2008; 10:1470–1476.

27. Tauro BJ, Mathias RA, Greening DW, Gopal SK, Ji H, Kapp EA, Coleman BM, Hill AF, Kusebauch U, Hallows JL, Shteynberg D, Moritz RL, Zhu HJ, et al. Oncogenic H-Ras reprograms Madin-Darby canine kidney (MDCK) cell-derived exosomal proteins following epithelial-mesenchymal transition. Mol Cell Proteomics. 2013; 12:2148–59.
28. Balaj L, Lessard R, Dai L, Cho YJ, Pomeroy SL, Breakefield XO, Skog J: Tumour microvesicles contain retrotransposon elements and amplified oncogene sequences. Nature Commun. 2011; 2:180.
29. Thakur BK, Zhang H, Becker A, Matei I, Huang Y, Costa-Silva B, Zheng Y, Hoshino A, Brazier H, Xiang J, Williams C, Rodriguez-Barrueco R, Silva JM, et al. Double-stranded DNA in exosomes: a novel biomarker in cancer detection. Cell Res. 2014; 24:766–769.
30. Kahlert C, Melo SA, Protopopov A, Tang J, Seth S, Koch M, Zhang J, Weitz J, Chin L, Futreal A, Kalluri R. Identification of double-stranded genomic DNA spanning all chromosomes with mutated KRAS and p53 DNA in the serum exosomes of patients with pancreatic cancer. J Biol Chem 2014; 289:3869–3875.
31. Lazaro-Ibanez E, Sanz-Garcia A, Visakorpi T, Escobedo-Lucea C, Siljander P, yuso-Sacido A, Yliperttula M. Different gDNA content in the subpopulations of prostate cancer extracellular vesicles: apoptotic bodies, microvesicles, and exosomes. Prostate 2014; 74:1379–1390.
32. Kanada M, Bachmann MH, Hardy JW, Frimannson DO, Bronsart L, Wang A, Sylvester MD, Schmidt TL, Kaspar RL, Butte MJ, Matin AC, Contag CH. Differential fates of biomolecules delivered to target cells via extracellular vesicles. Proc Natl Acad Sci U S A 2015; 201418401.
33. Cai J, Han Y, Ren H, Chen c, He D, Zhou L, Eisner GM, Asico LD, Jose PA, Zeng C. Extracellular vesicle-mediated transfer of donor genomic DNA to recipient cells is a novel mechanism for genetic influence between cells. J Mol Cell Biol. 2013; 5:227–238.
34. Cai J, Wu G, Tan X, Han Y, Chen c, Li C, Wang N, Zou X, Chen X, Zhou F, He D, Zhou L, Jose PA, et al. Transferred BCR/ABL DNA from K562 extracellular vesicles causes chronic myeloid leukemia in immunodeficient mice. PLoS ONE. 2014; 9:e105200.
35. Higginbotham JN, Demory BM, Gephart JD, Franklin JL, Bogatcheva G, Kremers GJ, Piston DW, Ayers GD, McConnell RE, Tyska MJ, Coffey RJ: Amphiregulin exosomes increase cancer cell invasion. Curr Biol. 2011; 21:779–786.
36. Koga K, Matsumoto K, Akiyoshi T, Kubo M, Yamanaka N, Tasaki A, Nakashima H, Nakamura M, Kuroki S, Tanaka M, Katano M: Purification, characterization and biological significance of tumor-derived exosomes. Anticancer Res. 2005; 25:3703–3707.
37. Atay S, Banskota S, Crow J, Sethi G, Rink L, Godwin AK. Oncogenic KIT-containing exosomes increase gastrointestinal stromal tumor cell invasion. Proc Natl Acad Sci U S A. 2014; 111:711–716.
38. Di Vizio D, Kim J, Hager MH, Morello M, Yang W, Lafargue CJ, True LD, Rubin MA, Adam RM, Beroukhim R, Demichelis F, Freeman MR: Oncosome formation in prostate cancer: association with a region of frequent chromosomal deletion in metastatic disease. Cancer Res. 2009; 69:5601–5609.
39. Meckes DG Jr, Shair KH, Marquitz AR, Kung CP, Edwards RH, Raab-Traub N. Human tumor virus utilizes exosomes for intercellular communication. Proc Natl Acad Sci U S A. 2010; 107:20370–5.
40. Demory BM, Higginbotham JN, Franklin JL, Ham AJ, Halvey PJ, Imasuen IE, Whitwell C, Li M, Liebler DC, Coffey RJ. Proteomic analysis of exosomes from mutant KRAS colon cancer cells identifies intercellular transfer of mutant KRAS. Mol Cell Proteomics. 2013; 12:343–355.
41. Putz U, Howitt J, Doan A, Goh CP, Low LH, Silke J, Tan SS. The tumor suppressor PTEN is exported in exosomes and has phosphatase activity in recipient cells. Sci Signal. 2012; 5:ra70.
42. Gabriel K, Ingram A, Austin R, Kapoor A, Tang D, Majeed F, Qureshi T, Al-Nedawi K. Regulation of the Tumor Suppressor PTEN through Exosomes: A Diagnostic Potential for Prostate Cancer. PLoS ONE 2013; 8:e70047.
43. Antonyak MA, Li B, Boroughs LK, Johnson JL, Druso JE, Bryant KL, Holowka DA, Cerione RA. Cancer cell-derived microvesicles induce transformation by transferring tissue transglutaminase and fibronectin to recipient cells. Proc Natl Acad Sci U S A. 2011; 108:4852–4857.
44. Melo SA, Sugimoto H, O'Connell JT, Kato N, Villanueva A, Vidal A, Qiu L, Vitkin E, Perelman LT, Melo CA, Lucci A, Ivan C, Calin GA, et al. Cancer Exosomes Perform Cell-Independent MicroRNA Biogenesis and Promote Tumorigenesis. Cancer Cell. 2014; 26:707–721.
45. Lai CP, Kim EY, Badr CE, Weissleder R, Mempel TR, Tannous BA, Breakefield XO. Visualization and tracking of tumour extracellular vesicle delivery and RNA translation using multiplexed reporters. Nat Commun. 2015; 6:7029. doi: 10.1038/ncomms8029.:7029.
46. Hoshino A, Costa-Silva B, Shen TL, Rodrigues G, Hashimoto A, Tesic Mark M, Molina H, Kohsaka S, Di Giannatale A, Ceder S, Singh S, Williams C, Soplop N, et al. Tumour exosome integrins determine organotropic metastasis. Nature. 2015; 527:329–35
47. Carmeliet P, Jain RK: Molecular mechanisms and clinical applications of angiogenesis. Nature. 2011; 19;473:298–307.
48. Hida K, Hida Y, Amin DN, Flint AF, Panigrahy D, Morton CC, Klagsbrun M: Tumor-associated endothelial cells with cytogenetic abnormalities. Cancer Res. 2004; 64:8249–8255.
49. Gunsilius E, Duba HC, Petzer AL, Kahler CM, Grunewald K, Stockhammer G, Gabl C, Dirnhofer S, Clausen J, Gastl G: Evidence from a leukaemia model for maintenance of vascular endothelium by bone-marrow-derived endothelial cells. Lancet. 2000; 355:1688–1691.
50. Ricci-Vitiani L, Pallini R, Biffoni M, Todaro M, Invernici G, Cenci T, Maira G, Parati EA, Stassi G, Larocca LM, De R.

Tumour vascularization via endothelial differentiation of glioblastoma stem-like cells. Nature. 2010; 468:824–828.

51. Mulcahy LA, Pink RC, Carter DR. Routes and mechanisms of extracellular vesicle uptake. J Extracell Vesicles. 2014; 3. doi: 10.3402/jev.v3.24641. eCollection; 2014.:10.

52. Christianson HC, Svensson KJ, van Kuppevelt TH, Li JP, Belting M. Cancer cell exosomes depend on cell-surface heparan sulfate proteoglycans for their internalization and functional activity. Proc Natl Acad Sci U S A. 2013; 110:17380–17385.

53. Fitzner D, Schnaars M, van RD, Krishnamoorthy G, Dibaj P, Bakhti M, Regen T, Hanisch UK, Simons M. Selective transfer of exosomes from oligodendrocytes to microglia by macropinocytosis. J Cell Sci. 2011; 124:447–458.

54. Braicu C, Tomuleasa C, Monroig P, Cucuianu A, Berindan-Neagoe I, Calin GA. Exosomes as divine messengers: are they the Hermes of modern molecular oncology? Cell Death Differ. 2015; 22:34–45.
